# Delivering Person-Centered Care During a Pandemic: Stakeholder Perspectives

**DOI:** 10.1093/geroni/igab046.1037

**Published:** 2021-12-17

**Authors:** Katherine Abbott, Kirsten Corazzini

## Abstract

Person-centered care (PCC) is an approach to care that both nursing homes (NH) and assisted living (AL) communities strive to provide. PCC is a philosophy that recognizes knowing the person and honoring individual preferences. However, when COVID-19 emerged, the NH and AL environments were ground zero for infection spread and disproportionate numbers of deaths among residents. As a result, many practices changed dramatically in efforts to reduce the transmission of COVID-19 in these communities. The purpose of this symposium is to discuss several projects that can speak to the impact of the pandemic on stakeholder efforts to provide PCC. First, Dr. Roberts presents feedback from residents and family members on the challenges COVID-19 created for family involvement in care conferences. In the second study, Dr. Behrens examines focus group data from direct-care nurses on their perceptions of delivering PCC related to risk of harm to staff and residents. The third study presents the voices of activities professionals who were implementing a PCC quality improvement project to communicate resident preferences, which illustrates both the importance of PCC during the pandemic, but also the challenges implementing during the pandemic. Fourth, the Kansas PEAK 2.0 program used provider feedback to direct and inform program responses through components such as consistent staffing. Finally, Dr. Zimmerman presents qualitative data from over 100 AL administrators, medical, and mental health care providers on their experiences pivoting during COVID-19. Our discussant will explore the implications of these studies in terms of the future of PCC in residential settings.

